# “You’re Going to Have to Think a Little Bit Different” Barriers and Facilitators to Using mHealth to Increase Physical Activity among Older, Rural Cancer Survivors

**DOI:** 10.3390/ijerph18178929

**Published:** 2021-08-25

**Authors:** Tamar Ginossar, Heidi Rishel Brakey, Andrew L. Sussman, Brittany Price, Miria Kano, Sally Davis, Cindy K. Blair

**Affiliations:** 1 Department of Communication and Journalism, University of New Mexico, Albuquerque, NM 87131, USA; 2 University of New Mexico Comprehensive Cancer Center, Albuquerque, NM 87102, USA; asussman@salud.unm.edu (A.L.S.); mkano@salud.unm.edu (M.K.); Ciblair@salud.unm.edu (C.K.B.); 3 Clinical and Translational Science Center, Health Sciences, University of New Mexico, Albuquerque, NM 87131, USA; hrishelbrakey@salud.unm.edu; 4 Department of Family and Community Medicine, University of New Mexico, Albuquerque, NM 87131, USA; 5 Gillings School of Global Public Health, University of North Carolina-Chapel Hill, Chapel Hill, NC 27599, USA; pbrittan@live.unc.edu; 6 Department of Internal Medicine, University of New Mexico, Albuquerque, NM 87131, USA; 7 Department of Pediatrics, University of New Mexico, Albuquerque, NM 87131, USA; sdavis@salud.unm.edu; 8 University of New Mexico Prevention Research Center, Albuquerque, NM 87131, USA

**Keywords:** aging, cancer survivors, physical activity, mHealth, rural, wearable activity trackers

## Abstract

Wearable activity trackers (WATs) hold great promise in increasing physical activity among older cancer survivors. However, older cancer survivors who reside in rural regions are at increased risk of being digitally marginalized. The goal of this study was to learn about WATs adoption motivation and needs of rural older cancer survivors who live in New Mexico, one of the most rural states with the lowest broadband Internet connectivity in the United States. We conducted six key informant interviews and recruited 31 older cancer survivors from rural counties statewide who participated in interviews and focus groups. Our results show great interest in using WATs as part of an intervention to alleviate barriers associated with the digital divide. Participants were offered diverse modalities to support them in adoption of the trackers. These results will be used to inform future interventions and policies focusing on increasing physical activity in older cancer survivors who reside in rural communities.

## 1. Introduction

The number of cancer survivors living in the United States (US) is growing. Estimated to be over 16.9 million in 2020, it is expected to reach 22.1 million by 2030 [1]. Most cancer survivors, defined as persons who were diagnosed with cancer from the time of diagnosis for the rest of their lives, are 60 years of age or older. Cancer survivors are at increased risk for comorbidities, but those who exercise have demonstrated improved physical health and overall quality of life [2]. Cancer survivors are less likely than the general population to meet the guidelines for physical activity [3]. Increasing older cancer survivors’ physical activity levels is a priority due to the potential to reduce their risk for comorbidities, improve their quality of life and even increase their longevity [4,5]. Interventions to increase physical activity among cancer survivors show promise as well as challenges to maintaining behavior change [6,7]. A shortcoming of previous research includes recruitment of a wide range of ages without examining the specific needs and challenges of older survivors [6,8]. Further, the majority of interventions have been conducted at academic medical centers and clinics and recruited cancer survivors who live in urban settings [9,10,11]. These studies documented the acceptability, potential efficacy and positive impact that such interventions can promote but fail to address needs of older cancer survivors living in rural areas. One of five cancer survivors live in rural regions. This group experiences disparities in cancer outcomes [12] and at least in some communities have lower rates of physical activity compared to their non-rural counterparts [13]. For these reasons, scientific reviews indicate the need for more research on physical activity interventions among cancer survivors [7], specifically those in rural regions [14,15]. Needs to be addressed in rural cancer survivor populations include more flexible interventions due to their limited financial resources [16], differences in the environment [17], limited transportation and access to gyms, fitness centers and other resources to be physically active [18,19].

Mobile health (mHealth) technologies, including consumer wearable activity trackers (WATs) and companion smartphone apps, have great promise in overcoming the limitations of in-person interventions to increase physical activity and reduce sedentary behaviors in older cancer survivors [20]. Studies revealed that WATs can provide behavioral modification strategies including self-monitoring, feedback, and goal setting, and that they are associated with behavior change in different populations [21,22,23], including cancer survivors [24,25,26]. Three recent reviews of studies that examined acceptability and impact of WATs on cancer survivors reported positive impact on behavior but called for additional research as a result of the limited number and scope of studies [26,27,28]. Moreover, studies did not target either rural or older cancer survivors [26,27,28]. Studies that examined acceptability of WATs in older adults documented the need to support their use of technology [20,29]. Recent pilot studies also show promise in WATs-based interventions among older rural individuals in Australia [30,31] and in Korea [32]. Rural populations in the US also experience inequitable access to technologies and increased barriers caused by under-resourced technological infrastructure [33], including WATs [30,31]. Therefore, it is likely that older cancer survivors who reside in rural communities experience increased barriers to technology adoption. Nevertheless, past studies did not explore the specific barriers and facilitators to using WATs to increase physical activity from the perspectives of older cancer survivors who reside in rural communities.

In designing mHealth interventions for this population, it is important to consider their unique needs and perspectives. There is a growing body of evidence that documents the importance of exploring individuals and groups that are members of more than one marginalized category [34], and to bring to the front their voices, complex identities and experiences of multiple social pressures [35], including those of older adults in rural communities [36,37]. It is, therefore, vitally important to explore the perceptions and experiences of older cancer survivors in rural communities, rather than infer from studies that previously conducted with individuals from other communities. The intersectionality of identities as people who are cancer survivors, older adults, and residents of rural areas increase these persons’ risk of being digitally marginalized. The digital divide is defined not only by physical access and connectivity to the Internet through ownership of computers, cellphones, and coverage through cellphone and broadband. It also includes digital literacy, which relates to users’ ability to navigate digital environments [38]. Thus, it is important to evaluate the acceptability of WATs, barriers to implementation, and the need for technical support in rural cancer survivors in diverse communities.

Limited application of theories constitutes a persistent gap in physical activity interventions in rural communities [39] and in mHealth interventions [40,41,42]. The diffusion of innovation (DOI) theory [43] provides a useful framework to consider the design and implementation of evidence-based interventions. According to the theory, individuals perceive the usefulness of innovations as a predictor of adoption. These perceptions relate to the degree to which innovations become embedded in larger social systems and networks making them accessible to recipients of the intervention. Hence, the adoption of innovations over time in a social system is determined by the characteristics of innovations and individuals’ resources and social positions. Five characteristics of an innovation determine its adoption: (1) relative advantage, the perception that an innovation is better than an earlier concept/innovation; (2) compatibility, the perception that an innovation is consistent with the needs, experiences, practices and social systems of a potential adopter; (3) complexity, how difficult or easy an innovation is to learn and use; (4) trialability—the extent to which a potential adopter can experiment with the innovation; and (5) observability—whether the results (benefits) of the innovation are visible by members of the social system. WATs could be adopted by older, rural cancer survivors as long as they perceive them as superior to other options, recognize that it meets their needs, can test its function, and learn and use it with ease. DOI has been used to explain older adult perceptions of technology [44], as well as older adult use of smartphone devices [45].

We conducted formative research in preparation for design and implementation of a community-engaged mHealth intervention that would utilize WATs as a tool to motivate and inform older cancer survivors in rural New Mexico (NM) to increase their physical activity. NM is a southwestern border state with tremendous cultural richness, unique history, and natural recreation areas. It is also one of the most rural, digitally and medically underserved states in the country [46]. Of its 33 counties, 32 are medically underserved [47] and all but one county have a below average health literacy level [48]. The state has large areas with limited cellphone coverage and is one of the three most digitally underserved states with growing rural/urban differences in bandwidth connectivity [49]. We anticipate that these factors (i.e., being medically underserved, under-resourced and digitally marginalized) interact to increase the disadvantage of this population and the unique challenges they face, as well as the strategies they have developed to overcome them. Hence, an important goal of this study was to examine the specific needs and contextual realities of our participants in ways that would inform future interventions, including our own.

The objective of this study was to examine the appeal and relevance of an mHealth intervention to increase physical activity for older, rural cancer survivors in NM. Specifically, to inform a future intervention, we sought to understand the barriers and facilitators to using WATs by older cancer survivors who live in rural NM communities, and the type of technical support they needed to facilitate a future mHealth intervention using WATs to increase physical activity.

## 2. Methods

This was part of a larger study that explored research recruitment barriers and facilitators, perceptions of physical activity, and acceptability of WATs among cancer survivors over the age of 60 who resided in rural communities in NM. Data collection took place from October 2017 through September 2018 following IRB approval (HRRC# 17-198).

### 2.1. Identifying Locations and Recruitment

Our goal was to select rural counties with wide representation across the state in terms of geographic location, cancer prevalence, social economic status, and ethnicity. We identified six rural counties using the rural-urban commuting area (RUCA) codes that classify US census tracts based on population density, urbanization, and daily commuting. The NM Tumor Registry provided estimates of the average annual cancer counts per county. We selected counties that had at least one town with population of 10,000–49,999 people (RUCA codes 4–6). See Table 1 for details about selected counties (for anonymity, we are not including the names of the counties and towns).

In the first phase of data collection, we identified key stakeholders who lived in our target counties. Key informant interviews are important in reaching underserved populations for community-based healthcare research [50], including in NM [51]. To be eligible to participate in key informant interviews, participants had to live in one of the six target counties and to hold a health-related position within their communities. We identified potential candidates through health councils and public health organizations. We conducted the interviews at a convenient time and location of their choosing and provided a $50 merchandise card as compensation for their time. Interviews lasted 60–90 min and centered on structural, systemic, and cultural barriers related to our research as well as specific strategies for recruiting local older cancer survivors. Their perspectives informed the interview guide and the recruitment methods we utilized in the second phase. To maintain confidentiality of the informants who are members of small communities, we only provide demographic information in an aggregated form with no specific occupational information.

Upon completion of interviews and qualitative analysis of the key informant interviews, we used snowball sampling to recruit older cancer survivors from rural counties. We followed stakeholders’ suggestions and reached out to individuals as well as organizations that may have direct contact with cancer survivors over the age of 60 (e.g., in cancer support groups, senior centers and community centers). We placed recruitment flyers and postcards in key community locations and advertised on the radio and in newspapers. Interested individuals contacted the research team; we explained the study and screened for eligibility. People eligible to participate were: (1) aged 60 or older, (2) had received a cancer diagnosis, (3) lived in one of the six identified counties, (4) lived on their own (not in an assisted living/skilled nursing facility), (5) were able to walk approximately 0.25 miles without an assistive device, (6) did not have any medical conditions that would make it very difficult or impossible to add light physical activity into their daily life, and (7) spoke, read, and understood English fluently.

In-person focus groups were conducted with older cancer survivors (*n* = 21) in three counties. Participants in the remaining three counties were unable to join a focus group. We therefore adapted our protocol to allow for telephone interviews (*n* = 10) that provided greater flexibility and participation. Informed consent was provided prior to participation. We collected demographics of age, gender, race, ethnicity, language spoken at home, and education level. Self-reported general health was assessed using the global general health question from the Medical Health Outcomes Study Short Form 36-item survey (SF-36, v2). This single question has good validity (r = 0.66) and reliability (ICC = 0.69) [52]. We asked participants what types of technologies they used, the support they would need to use a WAT, and interest in this type of project. Based on a previous study by the principle investigator with older cancer survivors in urban, clinic-based setting in NM [20], we were interested specifically in Fitbit for the future intervention. We passed around a Fitbit Alta HR for participants to examine and showed them screenshots of the app. When conducting telephone interviews, we described the WAT and the app. All participants received a $50 merchandise card as compensation for their time. All interviews were audio recorded and professionally transcribed.

### 2.2. Analysis

We analyzed transcripts and notes using an iterative, thematic approach [53] in NVivo 11 (QSR International). Thematic analysis is useful in examining perspectives of different participants [54]. The analysis was largely inductive. Two study team members jointly created a coding structure (HBR, BP), independently analyzed each transcript, and met frequently to discuss discrepancies and revisions, ultimately achieving consensus in their analyses. They each coded the remaining transcripts and met to discuss. Throughout this process, they periodically met with the full research team to discuss coding and gain input on analysis, themes, and areas of focus. Another author (TG) coded the transcripts independently, verified codes and introduced new ones, which were later discussed by the whole team in iterative processes before the final analysis received the team’s approval. For example, at the analysis phase the DOI theory emerged as relevant to participants’ experiences. We have incorporated DOI as a sensitizing concept [55,56]. Introducing theory at the analysis stage of a qualitative inquiry is consistent with a traditional approach to theoretical sensitivity in qualitative research first introduced by Glaser [57,58].

## 3. Results

Key informant interviews (Phase 1). We interviewed one key informant per target county (*n* = 6). Key informants were between the ages of 26 and 65 years, primarily female (*n* = 5, 83%), Hispanic White (*n* = 4, 67%) and college graduates (66.7%). These interviews were important in highlighting possible barriers to our intervention, including recruitment and implementation. In particular, these interviewees emphasized the digital divide, in the form of varying degrees of cellphone and Internet coverage and digital literacy at the different communities as significant barriers. For example, one interviewee explained that intervention strategies should be tailored to the specific needs of each community:

“In some of these areas there’s no service, no accessibility to broadband. Where you do have broadband, it’s not the best [and] there’s just people who can’t afford it. You’re going to have to think a little bit different. What you’re going to do in County [X] is going to be very different than what you’re going to do in County [Y].”

Key informants noted a lack of connectivity for older adults in their community. One of them shared: “My in-laws don’t have Internet access. My parents don’t have Internet access”. Another highlighted that having digital access does not ensure digital literacy: “[older cancer survivors] may have a smartphone, but there again, how much [do] they use it? Probably not a whole lot. Even my mother-in-law kind of struggles trying to get through it. I struggle through it sometimes”. The insights from the informants were valuable in recruitment and in gaining access to the different communities as well as in highlighting the digital connectivity challenges in their communities. This input informed the interview guide. For instance, we asked participants about preferences for community-based support based on suggestions from the key informants.

Participants’ focus groups and individual interviews (Phase 2). The 31 cancer survivors who participated in focus groups (*n* = 21) or individual interviews (*n* = 10) were between the ages of 60 and 89, primarily female (*n* = 23, 74%), Non-Hispanic White (*n* = 20, 65%), had a two-year post high school degree or less (*n* = 18, 58%), spoke only English in their homes (*n* = 27, 87%), and rated their general health as very good or good (*n* = 23, 74%; see Table 2).

Most participants were not familiar with the technology prior to our interactions and were interested in a future WAT intervention to increase their physical activity. However, only a few seemed confident in their ability to use the technology, due to their digital literacy, connectivity, or both. Therefore, the majority of our participants stated that they would need support before they could feel confident using WATs effectively. One woman summarized what most people said in the following statement: “Interested, yes. Whether or not I’d be able to do it, that’s a different [story]”. Although participants expressed strong motivation to use a WAT which they perceived as compatible with their goals, they were concerned about WATs’ complexity, and requested detailed, accessible instructions and support that would reduce confusion resulting from WAT complexity. These three themes of motivation, barriers, and requests for support are detailed in the following section using DOI framework.

### 3.1. Motivation to Use a WAT

As an overarching theme, participants felt that WATs use was compatible with their goal to be healthy. They described different motivators as reasons to adopt WATs as part of an intervention. These reasons included curiosity and desire to learn and try new things, perception that using a WAT would increase their motivation to be physically active through tracking and reminders, and that this technology has a relative advantage over other technologies and current practices (see Table 3 for breakdown of their perceptions following DOI’s characteristics of innovations).

Curiosity and novelty. Our proposed intervention to provide an opportunity to try the WAT at no cost increased the technology’s trialability. Some participants explained that they did not know much about WATs and expressed a general interest to try them without having specific expectations other than curiosity: “Looking forward to the benefits of using it. I don’t have one, so I have no expectations”, said one participant. The novelty of the WAT was perceived as a relative advantage, and participants expressed curiosity. Some expressed an interest in learning how to use the technology and in deepening their knowledge about their activity through tracking it: “I want to learn new things”, said another participant. Discussing the details of the proposed intervention and its mHealth features allowed some participants to reconsider previous decisions about rejecting adoption of WAT.

Increase motivation to be active. Others felt that a WAT would provide them with much needed motivation to be physically active: “It’s very helpful. It’s very motivating for me”, shared a participant. Another said: “I think that might be the accountability I’m looking for”. Others shared a hope that the WAT and the app would encourage them to be less sedentary and more active: “Get your a*s up, yeah”. In another group, a participant explained: “This week, I really sat on my… (laughter). Yeah, I think it would be a motivator”. In addition to increasing motivation by providing cues to action, participants mentioned that the tracking features of the WAT was important for awareness about one’s actual activity level, compared to their perceptions: “I think it puts it in perspective of what you were doing as opposed to what I’m interested [in doing]”, said a participant. Another participant compared the WAT tracking feature to journaling: “If it shows that I ain’t nothing but a couch potato [I will be motivated to exercise]—when you diet, the best form of a diet is to keep a journal. When you’re exercising, the same thing”.

In addition to participants’ expectations that the WATs would provide encouragement to move by alerting them about sedentary behaviors, they felt that tracking could be important in providing positive feedback on improvement for those who were new to physical activity, and for those who were trying a new form of activity. For example, one man considered purchasing a WAT prior to the study and said: “It’s a very, very good tool for someone that has been inactive to gauge how much they are improving”. Moreover, in discussing the WAT’s compatibility with their goals to keep their motivation to be healthy, participants listed different advantages that this technology held for them compared to other options, as discussed in the next section.

Relative advantage. Participants listed explicit advantages of the WAT over other technologies they were using. For instance, one participant viewed the WAT as having relative advantage in tracking her activity level: “Sometimes I’m motivated by a little bit of a goal […] I’m not really good at just writing on a piece of paper”. Other participants felt that the WAT had a relative advantage in providing detailed information. One participant noted: “on my smart phone they have a health app there that supposedly keeps track of how many steps you make in a day […] [the WAT] is a lot more detailed”.

In weighing the relative advantage of the proposed technology, participants felt that in addition to physical activity tracking, tracking nutrition, heart rate, sleep and other features were advantageous. They felt that these features can inspire diverse health-behavior changes: “that information can make you go ‘I should try eating more tomatoes,“ said one participant. Another discussed a heart condition and explained: “That [WATs] that you were talking about, that tracks your heartbeat or heart rate or whatever, that would be beneficial for me”. A participant explained that a WAT would provide more specific information about his blood pressure and other vital signs compared to the machines available in large stores: “I go to Sam’s or Walmart and I use their machines to check the blood pressure and such, but they’re still limited on what you can test compared to the WAT”. Another participant thought that she could benefit from tracking her sleep: “I thought when you were talking it wouldn’t be bad [to track] how much deep sleep I’m getting at night”.

Observability and use of WAT in social networks. Since WATs are worn on wrists, they are particularly observable to others. A few participants had friends or family members who used WATs and reported mixed experiences. Their perceptions of satisfaction impacted their own enthusiasm about possible adoption. For example, one woman who narrated a largely positive family attitude towards use of WAT mentioned that she was interested:

“My sister-in-law wears it and I think likes it very much. It’s a motivator for her. My son who’s a resident now, he had it for a while […] I notice people that are wearing these sort of elaborate [WATs] watch kinds of things, so it seems like it’s something that people are using. I would certainly, for me, recommend it. My husband will not use it.”

Others shared that individuals in their social networks were reluctant to use WATs or had negative experiences. They noted in particular that trackers did not have relative advantage to people who were already very active and motivated, as they did not need to increase their motivation or activity levels. One woman described her friends’ diverse reactions to use of WATs: “My neighbor has a WAT and she walks with it […]. One of my dear friends […] walks five miles every day and plays tennis and pickleball. It just wasn’t useful for her. It wasn’t a motivator”.

### 3.2. Barriers to Using WATs

Most participants perceived the WAT as complex, and potentially incompatible with the technological infrastructure available to them. These concerns included incompatibility of WAT due to (a) lack of physical access and connectivity, (b) digital literacy, and (c) other concerns about the relative advantage of this technology.

Digital access. Many participants were concerned that using a WAT and its app might not be compatible with their digital access. One interviewee shared: “Sometimes I’m in an area where there’s no electricity or cellphone coverage”. Connectivity was a major barrier in very remote counties, as one interviewee noted: “In those very most rural it’s primarily landlines, just because the Internet access and cellphone service isn’t very good”. However, cellphone coverage was an issue even in major towns. One town with almost 50,000 residents was described as providing intermittent cell coverage: “It’s not good if you’re looking to look at your emails or get online in some places in town”. A resident of a larger town with over 100,000 people said: “As soon as you pass the winery your service crazy improves. […] The mountains are blocking us”. In view of the limited connectivity even in larger towns, participants highlighted connectivity issues in more remote and frontier areas. For example, a participant advised:

“I don’t know if you know much about New Mexico, but County X is a really poor county. I mean, it’s like one of the five poorest counties in the US, so not a lot of people have smartphones. It is the modern world, so people do have Internet.”

However, not all our participants had Internet access, as one of our participants shared: “I don’t have a smartphone. Believe it or not, I still have a flip phone [...] I don’t have Wi-Fi here at home”. Participants’ description of their access often depicted a complex picture, in which their resiliency and resourcefulness allowed them to access some communication technologies intermittently by being creative and by tapping into resources in their social networks. For example, a participant who only had a flip phone shared that her son provided her with a tablet that she could use for the study, despite not having either a smartphone or Internet at home: “I think it’s a tablet or an iPad. I’m not sure what it is. When I go to my son’s house to take care of the kids, I can use it there”.

Digital literacy and perceived complexity of WATs. Concerns about their ability to use a WAT, sync it to the app on their smartphone or computer, and then using the app in a meaningful way dominated participants’ discourse. Participants highlighted that even having physical connectivity did not secure digital access due to limited digital literacy. One participant shared: “I have the computer and I’m spending a lot of time on it, but I still spend a lot of time not being able to figure out what the heck I’m supposed to be doing”. A participant explained her reluctance to use WATs in her experience with computers in general “Just hook it up to the computer sounds a whole lot easier than it turns out to be. Push button X is just fine, except you have push button X frequently gets you Y or Z or something else”. The terminology used to describe WATs was unfamiliar and increased participants’ uncertainty. For example, when the researcher asked, “What specific suggestions do you have for us to show you how to use the WAT and sync it with the app?” the participant responded: “See, that’s like talking Greek to me. All that you just said, I don’t know what any of that means”. Another person who was interviewed by telephone asked for clarifications about the WAT and noted “I’m sorry. I’d never heard it like that. They just call it a watch here”.

Privacy. Some individuals felt that the WAT would be incompatible due to concerns about privacy. These participants were attracted to the advantages they perceived, but were reluctant to have their information tracked. One man explained: “I am familiar with WATs. I’ve thought about [getting] them a couple of times. […] But I’m old school. […] it’s a little concerning to me about being tracked”. He listed his motivation to use WATs in his desire to keep healthy: “I might outgrow that in time because I’m not ready to go to the UNM Medical School yet”.

### 3.3. Instructions and Support

As a formative research, our analysis was intended to identify specific support modality that participants would need to use WATs successfully. In view of participants’ interest in adopting WATs, as well as the concerns about their ability to master the perceived complexity of accessing and navigating this technology, participants highlighted the importance of technical support, including provision of instructions in appropriate literacy level, provision of telephone support, and support within their personal networks.

Instructions. To mitigate the perceived complexity of WATs and increase digital literacy, participants highlighted the importance of simple, step-by-step instructions. They were open to different modalities, including instructions written at an appropriate literacy level with accompanying pictures, in a video, in person, or using a combination of these modalities. One person advised: “Just explain it really well. These people were our past accountants and our past researchers, so they’re by no means dumb. They’re just a little technologically challenged”. One of the participants that highlighted the importance of using appropriate literacy level in the instructions said: “It should definitely be in plain English, preferably with illustrations”. Some thought videos were a good idea, noting: “I think a video would be very helpful”, but others cautioned that videos could be too complex: “I don’t know if people listen to videos”. These different suggestions regarding the needs for diverse modalities all emphasized the importance of multiple strategies, clear messages and appropriate digital literacy rate.

Telephone support. Most participants thought that telephone support could be helpful, and had specific recommendations about best ways to provide such support. Some even liked the option of a combination of calls with texts or emails. A participant explained:

Telephone calls are nice because they’re more personal, and you can tell by a person’s voice and their inflection and everything what they’re thinking and feeling and how their personality is. Text messages are fine because I can always go back and read those over again.

One participant suggested checking in with post cards, saying:

“Or if you don’t want to give them a call, maybe send them a postcard and say, ‘Hey, you’re two months into this. Thank you for your help. I hope you’re doing great. If not, give me a call. If you’re having trouble with your technology, with your WAT. Is it working well?’”

Overall, participants seemed to share a preference to be contacted by the research team, and saw different modalities as acceptable, emphasizing the power of check-in, regardless of the format used to support.

Local support. Participants responded favorably to the idea of having some sort of local in-person support. A participant related that “if you could get a computer contact in this town you’re doing a lot better [than just telephone support by the team]”. Some participants suggested recruiting local resources:

“A few volunteers that might understand the community and might be willing to go up once a week or once a month, going out to the communities and checking in on people…You have the phone calls from UNM folks twice a week, and then you’re going to have folks coming up to your house and checking in on you twice a month.”

His answer emphasized the importance of the local touch and the need to supplement telephone calls with local support. Another man suggested that the local study participants would provide support to one another. “We’d probably be in contact with each other. I think that would probably help quite a bit, both in terms of technical support and moral support”. Many said they had family members that would help them with the technology. “If we can get the grandkids involved to help their grandparents […] we can create a good impact”. Some participants reported that they can rely on their social networks for support, mentioning spouses, adult children, friends and others. A participant said: “My sons are all pretty adept at that. I don’t think I would need a community member”. Another thought her friends could help: “I also have quite a few friends that have [WATs], so I’m thinking I could probably go hang out with a friend, too, if I needed help”.

In contrast, other participants reported that they did not have local support to rely on, either because they lived too far from town or because they did not know anyone who could provide assistance. For example, one woman shared: “I live so far from anybody that I sort of manage on my own”. Another person said, “I wouldn’t want to have to rely on [friends or family]. For one thing, […] I’ve only been in the place I’m living for four or five months. I do have friends there yes, but I don’t think they know anything more about the computer than I do”. Another participant shared: “I have no family members anywhere in New Mexico. My children are both dead. My brothers are all dead”.

Additionally, a few participants said they did not think they even needed extra support. One of them stated: “I think I can handle it. I’m 74, but I try to stay up with the times as much as possible. My wife, she’s now retired. She was a legal secretary. She’s pretty sharp, too”.

Group support. While our proposed intervention did not offer an explicit cancer survivorship support group, participants across the state expressed their interest in such a component. In one focus group, a participant shared that she needed: “more of a support group of people rather than a gadget”. Another interviewee in a different community echoed her thoughts: “Wouldn’t you like to talk to somebody about it? Just to someone that can empathize”. In another group, a person explained further, “I think it would be helpful just to give people a platform to talk about it. Most people, they have to talk about it with their friends and family who are concerned and generally invested [...] You don’t always get the best advice when you talk to your family and friends”. Our participants consistently emphasized a need for peer support to supplement mHealth interventions among older, rural cancer survivors.

## 4. Discussion

Although only a few of the rural, older cancer survivors who participated in this study expressed confidence in their ability to learn how to use WATs on their own due to the digital divide, our participants were interested in adopting a WAT as part of an intervention. Applying DOI to analyzing the results provided a framework through which to guide understanding of participants’ lived experiences and to structure future interventions. Participants felt that WATs were compatible with their interest in increasing their physical activity levels, and that they would provide a relative advantage over other technologies and practices by motivating them to be more active. Participants’ main concerns centered on WATs’ complexity, and many of their questions revolved about how difficult or easy it was to use these devices. Our methods allowed for some level of trialability, as we shared a WAT in focus groups, and we promised that the intervention would allow them to try the WAT free of charge. This proposed intervention was attractive to our participants. The WATs have potentially high observability, but only a few participants shared that they observed friends using the WATs.

These findings support previous studies that documented the importance of interventions to increase physical activity in cancer survivors [5,11,59,60]. They also are consistent with research that explored the specific needs and barriers facing cancer survivors in rural communities [16,31]. Similar to rural cancer survivors in Australia [61], our participants expressed the need for ongoing social support. These findings also align with recent studies that highlighted the importance of motivation in physical activity of cancer survivors [62]. Indeed, the use of qualitative methods to examine cancer survivors’ barriers and facilitators to physical activity elicit important insights from different communities [63]. Similarly, a growing body of research documented the acceptability of WATs in diverse communities including those who suffer from chronic illnesses [29], rural cancer survivors in Australia [32], and rural adults [64], including in South Korea [30]. However, our study was the first to focus on survivors who are both older and rural. Therefore, it advances the knowledge about intersectionality in identities of cancer survivors. Furthermore, this is the first study that centered on cancer survivors’ perceptions of WATs as a future intervention by applying the DOI. This theoretical perspective would facilitate future interventions that would be tailored not only to participants digital connectivity, but also to their perceptions of the technology.

Furthermore, participants’ points about the potential importance of WATs to increase their motivation to be physically active are consistent with studies on WATs use among cancer survivors and/or rural adults [26,27,30].

Due to the challenges of the digital divide, our participants stressed the importance of technical support along with varied preferences for support modalities. This notion echoed key informants’ advice about the need to tailor strategies for recruitment and implementation. Participants’ preferences were not only based on individual inclinations, but also on the specific resources available in different communities. In addition to technical support, they expressed a wish for groups to provide peer emotional and informational support. This finding is consistent with research on the relationship between social support and physical activity among older adults [65], and on the importance of social networks in reducing difficulty of online health information seeking [66]. As WATs have the potential to increase social support through sharing of the results [67], future interventions should incorporate social and peer support.

This study advances understanding of the applicability of DOI to mHealth promotion. In the case of older, rural cancer survivors, DOI-informed analysis revealed the complexity and diversity of participants’ perceptions regarding future adoption of a WAT to increase physical activity. In contrast to DOI that originally emphasized motivation of the elites to adopt innovations [68], our findings are important in understanding the motivation of marginalized individuals to adopt WATs as a new technology. Whereas barriers were acknowledged and would have to be addressed in future interventions, the resilience and resourcefulness of participants demonstrate how cancer survivor strength, perseverance, and openness to technologies that may increase their well-being may serve as a resource. Interventions should, therefore, be tailored to acknowledge the capacities as well as the needs of these individuals.

These findings underscore the importance of examining specific contexts in the lives of cancer survivors rather than operating off pre-conceived assumptions. For example, whereas WATs are readily observable in many settings, they were not common in the social circles of some participants, and therefore, were less familiar for some of those we interviewed. Even so, although often digitally marginalized, our participants perceived the WATs as having relative advantage over current technologies and practices.

Our study has important strengths, including working with a marginalized community and engaging both key informants and rural cancer survivors in NM in formative research to explore the feasibility and acceptability of using WATs to increase physical activity. We were able to recruit an under-represented population, with intersectional identities of older age, cancer survivorship, and rurality. Our sample was heterogenous in terms of race-ethnicity and educational levels. Additionally, considering the needs of our participants, using DOI as a theoretical framework can enhance the utility of these results in designing future interventions.

Naturally, our study has limitations. First, as a qualitative study that recruited using a convenience sample, our findings cannot be generalized to other cancer survivors in these or other rural counties. Similarly, inferences cannot be made about causality. For instance, based on our data we cannot determine whether certain demographics are associated with differences in perceptions or in support needs. Moreover, although we have utilized diverse recruitment methods that were guided by key informants, these methods introduced some selection bias. For instance, a cancer diagnosis carries stigma in some communities, and survivors in these communities might not be interested in coming forth and sharing their survivorship needs. Additionally, our team did not include local co-researchers from rural communities, and we did not measure participants’ levels of support.

Future research should expand the scope of this research by using larger surveys and outreach methods, such as contacting individuals based on cancer registry information and through local clinics to reach additional survivors. Studies should also explore how to reach individuals that are concerned about cancer stigma and to what degree they might be interested in using WATs to increase physical activity. In addition, researchers should dedicate additional resources to include co-researchers from local communities, and to recruit Native American as well as Spanish speaking survivors to learn about their information and support needs regarding use of WATs to increase physical activity. Finally, our participants’ interest in social support should be explored in future studies to better understand the possible link between their current levels of social support and expressed needs, as well as the ways in which such support can increase use of WATs and physical activity.

## 5. Conclusions

Despite experiencing digital marginalization, older cancer survivors in rural NM are interested in using WATs to increase their physical activity. In view of the limited connectivity and consequently low self efficacy and digital literacy, they request technical and social support that would allow them to use WATs effectively. Their motivation was based on curiosity and interest to try the new technology, as well as on their desire to maintain or increase their physical activity levels. These findings advance the knowledge on diffusion of innovations from the perspectives of community members who have been left behind. It shows that in the case of our participants, non-adopters were in fact interested in adopting this new technology. Therefore, barriers to adoption are predominantly structural. Therefore, there is a need for future interventions that would meet rural, older cancer survivors’ need for tailored interventions to increase physical activity in this specific population. Given the specific technological challenges and the need for peer support, it is important that future interventions will focus on this group of older cancer survivors who live in rural communities, rather than create “one size fits all” interventions with cancer survivors of different ages, or in rural settings. Such interventions can include the use of WATs, as long as the different barriers to digital connectivity are addressed.

## Figures and Tables

**Table 1:** Characteristics of Selected Counties.

State Quadrant	RUCA Codes	Population (Approximate)	Average Annual Cancer Diagnoses, per Capita
Northwest	4, 10	30,000	252
Northwest	1, 2, 6, 7, 8	130,000	184
Northeast	2, 4, 5	30,000	235
Southwest	4, 5, 10	30,000	369
Southwest	4, 5	30,000	379
Southeast	4, 5, 10	70,000	230

**Table 2:** Demographics characteristics of participants (*n* = 31) in interviews and focus groups of rural cancer survivors.

Participant Demographics		
**Age Group**	***n***	**%**
60–64	9	29%
65–69	6	19%
70–74	10	32%
75–79	3	10%
80–84	1	3%
85–89	2	6%
**Sex**		
Male	8	26%
Female	23	74%
**Race/Ethnicity**		
Hispanic	8	26%
White, Non-Hispanic	20	65%
American Indian	1	3%
More than One	2	6%
**Education**		
High School Graduate	3	10%
Some College	13	42%
Two-Year Degree	2	6%
Bachelor’s Degree	6	19%
Postgraduate Degree	6	19%
Unknown	1	3%
**General Health**		
Excellent	3	10%
Very Good	14	45%
Good	9	29%
Fair	4	13%
Poor	1	3%
**Total**	31	100%

**Table 3:** WAT Characteristics and Participant Quotes.

Characteristics of WAT	Sample Quotes
Relative Advantage	“On my smartphone they have a health app there that supposedly keeps track of how many steps you make in a day… [The WAT is] a lot more detailed.” “I go to Sam’s or Walmart and I use their machines to check the blood pressure and such, but they’re still limited on what you can test compared to the [WAT].”
Compatibility	“I don’t have a smartphone. Believe it or not, I still have a flip phone...I don’t have Wi-Fi here at home.” “I think it’s a tablet or an iPad. I’m not sure what it is. When I go to my son’s house to take care of the kids, I can use it there.” “I think I can handle it. I’m 74, but I try to stay up with the times as much as possible.”
Complexity	“I have the computer and I’m spending a lot of time on it, but I still spend a lot of time not being able to figure out what the heck I’m supposed to be doing.” “That’s like talking Greek to me. All that you just said [about syncing to an app], I don’t know what any of that means.”
Trialability	“I also have quite a few friends that have [WATs], so I’m thinking I could probably go hang out with a friend, too, if I needed help.”
Observability	“My neighbor [is] 73, maybe. She has a [WATs] and she walks with it. One of my dear friends…has a [WATs] and she didn’t like it.” “I notice people that are wearing these sort of elaborate [WATs] watch kinds of things, so it seems like it’s something that people are using.”

## Data Availability

Aggregate data may be available for research purpose upon reasonable request to the senior author (C.K.B.).
